# Expression of Putative Immune Response Genes during Early Ontogeny in the Coral *Acropora millepora*

**DOI:** 10.1371/journal.pone.0039099

**Published:** 2012-07-06

**Authors:** Eneour Puill-Stephan, François O. Seneca, David J. Miller, Madeleine J. H. van Oppen, Bette L. Willis

**Affiliations:** AIMS@JCU, James Cook University, Townsville, Queensland, Australia; 2 ARC Centre of Excellence for Coral Reef Studies and School of Marine and Tropical Biology, James Cook University, Townsville, Queensland, Australia; 3 Australian Institute of Marine Science, Townsville, Queensland, Australia; 4 Laboratoire Optimisation des Régulations Physiologiques, Université de Bretagne Occidentale, Brest, France; 5 ARC Centre of Excellence for Coral Reef Studies and School of Pharmacy and Molecular Sciences, James Cook University, Townsville, Queensland, Australia; 6 Department of Biological Sciences, Hopkins Marine Station, Stanford University, Pacific Grove, California, United States of America; University of Washington, United States of America

## Abstract

**Background:**

Corals, like many other marine invertebrates, lack a mature allorecognition system in early life history stages. Indeed, in early ontogeny, when corals acquire and establish associations with various surface microbiota and dinoflagellate endosymbionts, they do not efficiently distinguish between closely and distantly related individuals from the same population. However, very little is known about the molecular components that underpin allorecognition and immunity responses or how they change through early ontogeny in corals.

**Methodology/Principal Findings:**

Patterns in the expression of four putative immune response genes (apextrin, complement C3, and two CELIII type lectin genes) were examined in juvenile colonies of *Acropora millepora* throughout a six-month post-settlement period using quantitative real-time PCR (qPCR). Expression of a CELIII type lectin gene peaked in the fourth month for most of the coral juveniles sampled and was significantly higher at this time than at any other sampling time during the six months following settlement. The timing of this increase in expression levels of putative immune response genes may be linked to allorecognition maturation which occurs around this time in *A.millepora*. Alternatively, the increase may represent a response to immune challenges, such as would be involved in the recognition of symbionts (such as *Symbiodinium spp.* or bacteria) during winnowing processes as symbioses are fine-tuned.

**Conclusions/Significance:**

Our data, although preliminary, are consistent with the hypothesis that lectins may play an important role in the maturation of allorecognition responses in corals. The co-expression of lectins with apextrin during development of coral juveniles also raises the possibility that these proteins, which are components of innate immunity in other invertebrates, may influence the innate immune systems of corals through a common pathway or system. However, further studies investigating the expression of these genes in alloimmune-challenged corals are needed to further clarify emerging evidence of a complex innate immunity system in corals.

## Introduction

Many marine invertebrates lack a mature allorecognition system in their early life history stages, but are able to distinguish between self and non-self within days to months. For example, allorecognition (the ability to distinguish between self and genetically distinct members of the same species) reaches a mature state within the first two weeks after metamorphosis in the hydrozoan *Hydractinia symbiolongicarpus* [1], but requires more than two weeks in the bryozoan *Celleporella hyalina* [2], and up to four months post-settlement for the corals *Stylophora pistillata* [3] and *Seriatopora* spp. [4]. The lack of an efficient allorecognition response is likely to be universal among juveniles of both scleractinians (hard corals) and alcyonaceans (soft corals) [4], [5]. In support of this hypothesis, recent observations of fusion among juveniles of the coral *Acropora millepora* following aggregated larval settlement, as well as after contact through growth for up to 11 months post-settlement for full siblings, imply that this broadcast spawning coral species lacks a mature allorecognition system in its early life [6]. Moreover, some juveniles derived from fused aggregations of full siblings remained alive for almost two years, with no obvious signs of rejection among interacting genotypes. Genetic analysis confirmed that these young colonies were chimeras (i.e., different genotypes within the same colony, [6]). For colonies arising from fusions among non-siblings, the first signs of rejection were observed two months post-settlement and no fusion were observed after 3 months for non-siblings and after 5 months for half-siblings, suggesting that juveniles *of A. millepora* are unable to recognize genetically distinct conspecifics prior to two months and that the allorecognition response is maturing between 3 and 5 months [7].

Allorecognition in invertebrates is generally assumed to rely on pathways involved in innate immunity [8], thus PAMPs (pathogen-associated molecular patterns) may be involved in activating innate immune responses in corals. Such detection is believed to be achieved by proteins called Pattern Recognition Receptors (PRRs), which are expressed by innate immune cells and have the ability both to recognize PAMPs and to subsequently initiate an immune response [8]. However, the mechanisms of innate immunity in corals are, as yet poorly characterized and the link between allorecognition and detection of PAMPs is tenuous. Additionally, one study has suggested the existence of an adaptive alloimmune response in corals rather than an innate immune response [9]. Known PRRs include C-type lectins [10] and recent studies have highlighted their potential involvement in immunity, allorecognition and non-self recognition (the ability to distinguish between self and other species, as well as genetically distinct members within the same species) in corals [11], [12], [13], [14]. Coral lectins bind to glycans on the surface of *Symbiodinium* cells, the endosymbiotic microalgae that form an essential association with corals [14], and intact glycans have been shown to be crucial for the recognition and successful acquisition of *Symbiodinium* in the coral *Fungia scutaria* [14]. Thus lectin type genes are good candidate genes for molecular studies of non-self recognition and allorecognition in corals. Consistent with a role in immunity and potentially non-self recognition, the mannose-binding lectin gene in *A. millepora,* Millectin, has been shown to bind to various types of bacteria and to *Symbiodinium* cells [13]. Similarly, CELIII lectin homologs described in the sea cucumber *Cucumaria echinata* [15] are able to recognize cell surface carbohydrate chains on non-self tissues (i.e., PAMPs), and are thought to cause cell lysis by forming ion permeable pores in membranes after conformational change and oligomerization. High sequence similarities between the *A. millepora* CELIII predicted peptides (CELIII A036-E7 & A049-E7) and the CELIII described in *Cucumaria echinata* suggest that they may play similar functions [11]. Additionally, CELIII lectins have recently been shown to be under positive selection, which adds further weight to the suggestion that they are involved in immunity and/or allorecognition [16]. Moreover, lectins have been linked to complement C3 in the innate immune system of a solitary ascidian [17], and are potentially involved in a lectin-dependent complement system [17]. Coral complement C3 [18], [19] could be an important gene in allorecognition, with its activation relying on lectins, as described in ascidians [17]. Finally, coral apextrin encodes a protein that contains a membrane attack complex/perforin (MAC/PF) domain [19] and may also be involved in allorecognition.

To evaluate the roles of putative immune response genes in coral innate immunity and allorecognition, expression levels of lectins, complement C3 and apextrin were compared during early ontogeny (from one to six months post-settlement) in juveniles of the coral *Acropora millepora* in the field.

## Results

**Figure 1:**
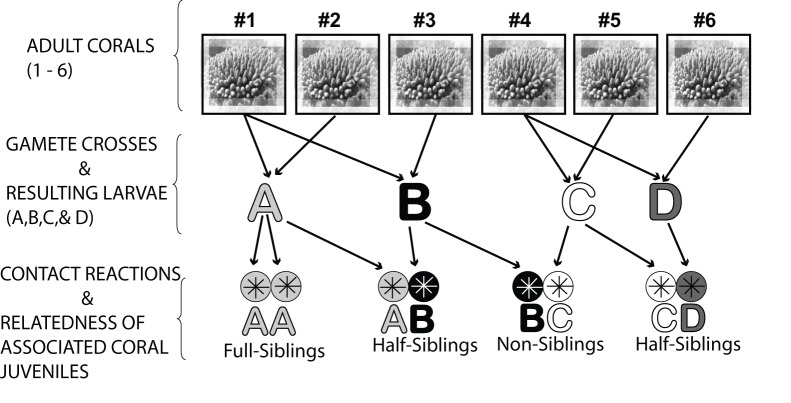
Design of experimental gamete crosses. Design of experimental gamete crosses involving six parent colonies of *Acropora millepora* (identified by a number #) that were performed to establish four larval cultures referred to as full sibling groups A, B, C, & D.

Using quantitative real-time PCR (qPCR), we examined the expression of four putative immune response genes (apextrin, complement C3, and two CELIII type lectin genes) in juvenile colonies of *Acropora millepora*. Four gamete crosses were performed (Figure 1), giving rise to four different juvenile groups named A, B, C and D. Gene expression was examined in each juvenile group which were sampled throughout a six-month post-settlement period.

### Internal Control Genes (ICGs)

Using GeNorm, the three best ICGs for normalization of gene expression were determined for each full sibling group (i.e., genotype). The ICGs used were: RpS7 (ribosomal protein S7) and RpL13 (ribosomal protein L13) for all four groups, plus GAPDH (glyceraldehyde 3-phosphate dehydrogenase) for group A, RpL9 (Ribosomal protein L9) for groups B and C, and Ctg1913 (Contig 1913: unknown transcript) for group D samples.

### Genes of Interest (GOIs)

Comparison of gene expression patterns through time using ANOVA for the factors SAMPLE(GENE*TIME) revealed that gene expression varied significantly over time in each of the four full sibling groups: full siblings A (SS = 0.21, F = 27.62, df = 39, p<0.01), full siblings B (SS = 0.09, F = 23.18, df = 25, p<0.01), full siblings C (SS = 0.33, F = 57.26, df = 32, p<0.01), and full siblings D (SS = 0.15, F = 14.49, df = 40, p<0.01).

**Figure 2:**
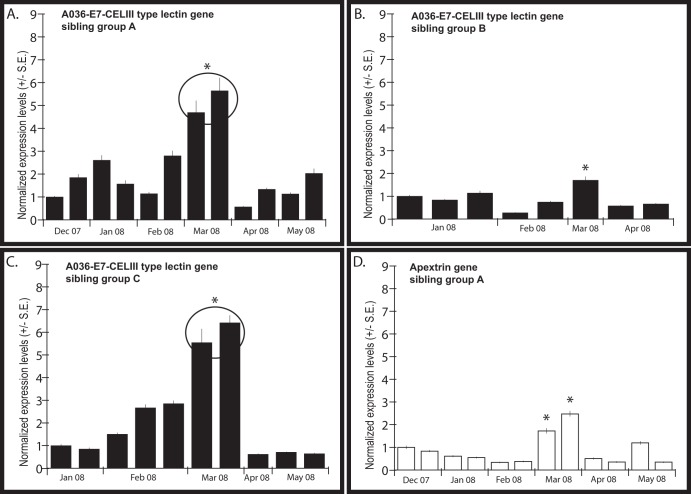
Gene expression results. Comparison of mean (±SE) normalized expression levels per biological sample (20–30 pooled polyps) for the genes A036-E7, a CELIII type lectin gene, (black histograms) or apextrin (white histograms), from December 2007 to 6 months post-settlement (May 2008) for: **A**) full sibling group A; **B**) full sibling group B; **C**) full sibling group C; **D**) full sibling group A. Each bar represents the averaged normalized expression levels for technical replicates (normalization performed with 3 ICGs). Samples with low mRNA concentrations (<8.75 ng. µL^−1^) were not used and consequently some biological replicates (max n = 3/time point) and/or time points (max 6 time points) are missing from the analysis and the figure.*: significant difference with all other samples (P<0.05, Tukey HSD). Circles: no significant difference between sample replicates for the same time point, when significant differences were observed with other time points.

Post-hoc Tukey HSD tests revealed that gene expression levels of one CELIII lectin gene (A036-E7) were significantly higher in March (i.e., 4 months post-settlement, Figure 2A, 2B and 2C) than at any other time for full sibling groups A, B, and C (p<0.01). Post-hoc Tukey HSD tests detected no significant difference in expression levels of the gene A036-E7 between samples within the full sibling groups A, B, and C in March (p>0.98).

Expression levels of apextrin for full sibling group A were also significantly higher in March (Figure 2D) than at all other sampling times in the six months post-settlement (p<0.01), but samples at the same time point differed significantly from each other (p = 0.037).

## Discussion

Juveniles of the coral *Acropora millepora* showed differential expression of putative immune response genes during a six month period following settlement, raising the possibility that the candidate genes tested may be involved in the maturation of allorecognition during early life history stages of corals. The most striking result was that expression of one of the CELIII type lectin genes, A036-E7, was significantly greater during the fourth month post-settlement in three of the four full sibling groups tested. CELIII type lectin genes are known to be involved in non-self recognition in a number of marine invertebrates [15], and implications that they are involved in immunity and/or allorecognition have recently been reinforced by evidence of positive selection [16]. Evidence that *A. millepora* juveniles from the same larval cohort showed the first signs of rejection in contact reactions at two months after settlement (January 2008 [7]) indicates that allorecognition systems are non-functional in *A. millepora* prior to two months. Although fusions ceased to occur between non-sibling juveniles growing into contact after two months, it took five months before fusions between half-siblings ceased [7], suggesting that maturation of allorecognition responses involved in distinguishing half-siblings requires approximately five months. The peak in expression levels of the CELIII type lectin gene at four months post-settlement provides corroborative evidence that it is likely to be involved in allorecognition in *A. millepora*.

The two-month delay in peak expression for the CELIII type lectin when compared to the appearance of the first signs of rejection between corals in contact reactions [7] might reflect a potential dual role for CELIII lectins in *A. millepora*. For example, the CELIII type lectin isolated from the sea cucumber *Cucumaria echinata* is involved in both the recognition of cell surface carbohydrate chains (i.e., PAMPs) and the lysis of non-self tissues [15], [20]. Such a dual role is possible because CELIII consists of 3 domains: the N-terminal carbohydrate binding domains (1 and 2) and the C-terminal domain (#3) [15], [20]. The N-terminal region binds to specific carbohydrates for recognition [15], which then triggers a conformational change in the C-terminal region, inducing oligomerization and resulting in the formation of ion-permeable pores on the target cell membrane [15]. Up-regulation of CELIII A036-E7 and A049-E7 at four months post settlement, rather than two months when the first signs of allorecognition were observed in *A. millepora* [7], may correspond to an increase in proteins required for the lysis of non-self tissues or cells. In support of this, high sequence similarities between the CELIII *Acropora* proteins (CELIII A036-E7 & A049-E7) and the CEL III described in *Cucumaria echinata* suggest that they may have conserved roles [11]. Consequently, the peak in the expression of the CELIII type lectin A036-E7 observed at 4 months post-settlement might be related to maturation of mechanisms involved in the recognition of half-siblings and/or the lysis of non-sibling tissues, both of which are consistent with the hypothesis that lectins play a crucial role in coral allorecognition.

Although three out of four full sibling groups displayed the same peak at 4 months post-settlement, we observed relatively high variability in gene expression levels among sibling groups (see Figure S1). Such variability is unlikely to be due to microhabitat or diurnal gene expression variation because all juveniles were grown at the same site and sampled at the same time of the day. It is also unlikely that the variability observed reflects only genotypic differences in expression patterns within the juvenile groups studied, which each had a unique set of genotypes, sharing only one parent at most (A with B, and C with D: half-siblings). However, it is probable that genetic differences among each sibling group also contributed to the variation in gene expression observed, as shown in studies of adult colonies of *A.millepora* that have highlighted high inter-genotypic variability in gene expression levels [21], [22].

It is important to note that the expression of CELIII type lectin drops after the peak in the fourth month. If up-regulation of this gene was associated only with immuno-competence, one would expect the levels of lectins to remain high after the fourth month, rather than drop to levels similar to those in the pre-competency period.

Therefore, in addition to enhanced immuno-competence, the peak and then the drop in expression of lectins might be linked to strong non-self recognition challenges occurring around four months post-settlement. We hypothesize that the initiation of non-self recognition and allorecognition maturation (∼2 months) could be followed by the recognition of incompatible symbionts, either *Symbiodinium* types and/or coral-associated bacteria which could have triggered the lectin up-regulation at four months. Studies on the establishment of coral-*Symbiodinium* symbioses reveal that a lack of specificity in the initial *Symbiodinium* uptake is followed by a winnowing of *Symbiodinium* types present within coral juveniles [23], [24]. Similar observations have been made for coral-associated bacteria, with coral juveniles hosting different and more variable bacterial communities than adults [25]. Although, we did not quantify *Symbiodinium* content, all recruits used in this study had brown pigmentation, indicating that they had acquired algal endosymbionts. Consequently, variation in gene expression of putative immune related genes could possibly be linked to a winnowing of algal and/or bacterial endosymbiont diversity rather than the presence or absence of endosymbionts.

During the first few months post-settlement, juveniles of *Acropora tenuis* and *A. millepora* are able to take up various *Symbiodinium* types, following which they become dominated by one type, although the period of winnowing may be quite extensive [23]. Lectins are suspected to play a role in the recognition of *Symbiodinium* in corals [14] and the temporal expression pattern observed here for CEL III A036-E7 is consistent with this notion. For symbiont recognition and successful acquisition, intact glycans on symbiont cell surfaces must bind to coral lectins [14]. Additionally, the ability of lectins to recognize various types of PAMPs on non-self was described for Millectin, a mannose binding lectin gene in *A. millepora* [13]. The binding region of this functional mannose binding lectin shows high sequence diversity, suggesting that Millectin may recognize and bind to various bacterial species and *Symbiodinium* types [13]. Furthermore, lectin-type genes show high levels of expression in alloimmune challenged ascidians [26], with the highest expression levels occurring when signs of rejection appeared between incompatibly paired ascidians. Ascidians potentially have a lectin-based opsonisation system (non-self tissues marked with lectins for destruction), with lectins playing a role in opsonisation at the point of rejection [26].

Lectins have also been linked to complement C3 in the innate immune system of a solitary ascidian and are potentially involved in a lectin-dependent complement system [17]. Although links between lectins and complement genes have not been demonstrated in corals, the presence of complement C3 [18], [19], in combination with the involvement of lectins in coral immunity [13], suggests the possible involvement of a lectin-dependent complement system in allorecognition pathways of corals. Although expression of complement C3 did not differ significantly throughout the 6 month period, it was highly expressed in the fourth month in juveniles of sibling group A, when both apextrin and CELIII type lectin A036-E7 were also highly (and significantly) expressed. The co-expression of these three genes during development raises the possibility that they may influence the immune system of corals through a common pathway or system. Further studies investigating the expression of apextrin, complement C3 and lectin-type genes in alloimmune challenged corals are needed to further elucidate the complex system of innate immunity that is emerging for corals. Other complement genes, such as complement C2 and C5 recently identified (based on ESTs and BLAST hits only) in *Aiptasia*, could also be relevant key genes in cnidarian immunity [27].

Our results add further questions to the on-going debate about links between allorecognition and immunity in colonial marine invertebrates. Some authors suggest that mechanisms of immunity should not be linked with allorecognition [28], and others argue more forcibly that allorecognition is likely to involve phenomena independent from innate immunity [29]. Although more studies are clearly needed, it seems likely that immunity and allorecognition share similar signalling and effector pathways, as suggested by the sequence similarity of putative immune genes in corals when compared to vertebrates [19].

To summarize, we show that one CELIII type lectin, A036-E7, was expressed at significantly higher levels four months post-settlement in juveniles of the coral *A. millepora*. Together with findings from recent studies of adult *A. millepora*, these results suggest that lectins are likely to play a crucial role in allorecognition and more generally in the innate immune system of corals, which is likely to have greater complexity than is commonly thought. We hypothesize that high expression levels of A036-E7 were linked to allorecognition maturation and/or non-self recognition challenges, such as algal symbiont recognition and the winnowing of algal symbiont diversity. Some caution should be exercised in interpreting these results, as peaks in the expression of genes studied were followed by a drop, while high expression levels would be expected to continue if allorecognition and immuno-competence were the only explanations for the observed peak. Future studies focusing on allorecognition and non-self recognition in “immune challenged” juveniles are required, for example by contact reactions or symbiont infection, in order to validate the roles of CELIII type lectins, complement C3 and apextrin in allorecognition and non-self recognition responses of corals.

## Materials and Methods

### Obtaining & Rearing Larvae

Mature colonies of *Acropora millepora* were collected from Nelly Bay, Magnetic Island, in the central Great Barrier Reef prior to the predicted spawning in October 2007. Colonies were transferred into 1000 L aquaria with running, temperature-controlled (28.5°C), 1 µm filtered sea water (FSW) at the Australian Institute of Marine Science. The genotype of each colony collected was determined based on analyses of 3 microsatellite loci, Amil2_006, Amil5_028, and Amil2_022 [30], prior to spawning to ensure corals were genetically distinct and to avoid crosses between clone mates. All coral collections were performed under Great Barrier Reef Marine Park Authority permit # G07/22554.1.

On the anticipated night of spawning, colonies were isolated in individual 70 L aquaria filled with FSW, and kept isolated until at least 45 minutes after spawning had commenced. Gametes were collected from the water surface and mixed with gametes from a second colony in separate 70 L fertilization aquaria filled with FSW. Four gamete crosses were performed, giving rise to four different gamete cultures (Figure 1). Gametes were allowed to fertilise for at least 1.5–2 hours, after which a small subset of eggs was sampled for microscopic confirmation of fertilization and development. Embryos were cleaned by three consecutive water changes of the 70 L fertilization aquaria, draining ∼90% of the water from the bottom and slowly refilling from the top each time. Embryos were then transferred into 500 L settlement aquaria supplied with running (1 L. min^−1^), temperature-controlled (28.5°C) FSW in a controlled environment facility, at a density of approximately one larva per mL. One 500 L settlement aquarium was established for each of the larval cultures A-D, thus each culture derived from one set of parents and comprised full sibling larvae (Figure 1). Embryos were checked microscopically in order to assess their development until ∼48 hours following fertilisation, when the fully developed, ciliated planula stage was reached. Once swimming larvae were elongated and had started to search the substratum for settlement sites, the bottom of each tank was covered with 30 autoclaved terracotta tiles (15 cm x 15 cm).

Eleven days after spawning, the laboratory-reared juvenile corals that had settled on the terracotta tiles were placed in the field. The terracotta tiles were tagged and skewered on rods through a hole in the centre of the tiles, with spacers (2–3 cm long) placed between each tile. The rods were suspended horizontally between two star pickets that had been driven into dead substratum at 5 m depth in Nelly Bay (Magnetic Island), an inshore reef [31], [32] with a gentle slope down to approximately 10 m.

To compare expression of immune genes throughout larval and juvenile development, 3 samples (20–30 polyps per sample) from each larval batch (A, B, C, and D) were collected at six time points between spawning and six months post-settlement. Sampling was performed one month after spawning (December 2007) and every month to May 2008. Each sampling was performed at midday (between 11∶00 and 13∶00 hours) using sterile scalpel blades. Each sample consisted of approximately 20–30 polyps per cryotube which was immediately snap frozen in liquid nitrogen and subsequently kept at −80°C. Three replicate tubes of samples were collected for each sibling group of juveniles (i.e., A, B, C & D) per time point (3×4 = 12 samples per sampling time). Consequently, every “sample” represented a pool (20–30 individuals per sample) of closely related coral juveniles defined as full sibling groups (see Figure 1). Therefore, for ease of reference, larvae and juveniles from tank A are referred to as full siblings A, larvae from tank B are as full siblings B, and so on (see Figure 1).

### mRNA Extraction, Treatment and cDNA Synthesis

Messenger RNA (mRNA) was extracted from coral samples using the Dynabeads® mRNA direct kit (Invitrogen), following the protocol detailed in [21]. mRNA was eluted in 10 mM Tris-HCl (pH = 7.5), and kept at −80°C. Following extraction, mRNA was treated for genomic DNA contamination using Turbo DNA free™ (Ambion). Then, mRNA concentration was estimated using a NanoDrop ND1000 (Nanodrop technologies). Finally, the quality of the mRNA and the ribosomal RNA (rRNA) contamination was verified using a 2100 BioAnalyzer (Agilent). High integrity in mRNA sample was represented by a curve with a steep left-hand slope (small size mRNA) and a gradual right-hand slope ending with a trail (large size mRNA). For samples used in this study, all curves showed good integrity. Subsequently, mRNA concentrations were re-calculated by taking into account rRNA contamination.

Complementary DNA (cDNA) synthesis was performed using SuperSript™ III first-strand synthesis supermix (Invitrogen). Samples for cDNA synthesis were prepared using a CAS 1200 robot (Corbett Robotics) by adding 70 ng of mRNA per reaction, 10 µL of 2× RT reaction mix, 2 µL of RT enzyme mix, and DEPC treated water (Ambion) in order to reach a final volume of 20 µL per reaction. Samples with low mRNA concentrations (<8.75 ng.µL^−1^) were not used for cDNA synthesis. Consequently, because of low mRNA concentrations, some biological replicates and/or time points were missing in the analysis. The cDNA synthesis was performed on a Gradient Mastercycler (Eppendorf) and the protocol consisted of the following steps: 25°C for 10 min, 50°C for 30 min, 85°C for 5 min, and the reaction was terminated by placing tubes on ice. All samples were prepared and processed during the same cDNA synthesis run. Subsequently, samples were diluted in DEPC treated water (Ambion) in order to reach a final concentration of 0.5 ng.µL^−1^, and were kept at −20°C.

### Primer Design

Primers were designed using OligoPerfect™ designer (Invitrogen), using the following parameters: primer size from 18 to 27 bases, melting temperature from 68 to 72°C, primer GC content from 50 to 57%, and amplicon size from 148 to 152 bp. Subsequently, melting temperatures, primer quality and primer dimer were assessed using the primer test section of FastPCR (http://www.biocenter.helsinki.fi/bi/Programs/download.htm).

Selection of candidate Internal Control Genes (ICGs) for normalization of gene expression was based on previous studies of gene expression in *A. millepora* [22], and primers were designed for: ribosomal protein genes S7, L13 and L9 (RpS7, RpL13 and RpL9 respectively), Ctg1913 and GAPDH (Table 1). Genes of interest (GOI) were chosen because of their suspected involvement in *A. millepora* immunity [11], [13], [16], [19], and primers were designed for apextrin, complement C3, and two CELIII type lectins: A036-E7, and A049-E7 (Table 1). Primers (Sigma-Genosys) were then re-suspended in 1×TE in order to reach a concentration of 200 µM. Subsequently 10 µM aliquots were made for every primer by diluting primers in DEPC treated water (Ambion).

**Table 1:** Genes and Primers.

Gene name	Primer sequence	Amplification region	Tm (°C)	GC (%)
RpS7*	F: t c t t c c c t g c c a c a t c a a c c t c c t	503–631	F: 70.4	F: 54.2
	R: g a a a c c c c a a g a t g c g g g t g a a c		R: 69.8	R: 56.5
RpL13*	F: a c t a t g c g g g c a a c g g a t g g t t c	94–244	F: 70.4	F: 56.5
	R: g g a t g g a g c a c g g a a a t g a a a t g g		R: 67.5	R: 50.0
RpL9*	F: g c c g c a t t c t c a c a c g c c t a a t g	278–427	F: 69.6	F: 56.5
	R: t g a t c a a g g g g g t c g t c t a t g g c t a		R: 69.1	R: 52.0
Ctg1913*	F: a g a t t g t g g c g t t g g g g a a t g c t	206–356	F: 70	F: 52.2
	R: c g c a c a g a a g c a g c a a g c a a t g a		R: 69.4	R: 52.2
GAPDH*	F: t g t t c c a a a g a a g c g c g c a t a a c c	1066–1213	F: 69.1	F: 50.0
	R: t t c c c t g g g a g a a g t t c g g t g g a		R: 70.2	R: 56.5
Apextrin	F: c g g g a c g c a a a c g t t t t g g a g t t	1915–2062	F: 69.4	F: 52.2
	R : c a g g a a a c a t c t t c g g g g c c a a c		R: 69.6	R: 56.5
Complement C3	F: t c a a g t g g a a g g t c g c g t g g a a a	199–351	F: 69.3	F: 52.2
	R: g c c t c c t t t t g g a a c c g g a a g t g		R: 69.5	R: 56.5
A036-E7	F: c t c a t t g c a t t g c t g g g g t c c t g	44–194	F: 69.6	F: 56.5
	R: t t g a g a g g c t g c t g t g g g g a a g a		R: 70.7	R: 56.5
A049-E7	F: t g t c c g a g g a t g c a t g t g g c a a t	1380–1530	F: 69.6	F: 52.2
	R : g c a a t c c t c a t c c a g g c a t c g t g		R: 69.2	R:56.5

Internal Control Genes (ICGs), Genes of Interest (GOI) and associated primers used in qRT-PCR reactions.

* Internal Control Genes for *Acropora millepora* [28].

### Quantitative Real-time PCR

GOIs and ICGs were amplified in 20 µL reactions (3 replicates/sample/gene), each containing 10 µL QuantiTect SYBR green (Qiagen), 6 µL DEPC treated water (Ambion), 1 µL Forward primer (at 10 µM), 1 µL Reverse primer (at 10 µM), and 2 µL of cDNA (at 0.5 ng.µL^−1^). All reactions were prepared using a CAS 1200 robot (Corbett Robotics), in order to minimize pipetting error. qPCRs were performed on a Rotor Gene 3000A (Corbett research), with the following steps: 15 min at 95°C, followed by 50 cycles of 10 sec at 95°C and 120 sec at 65°C, with a final melting step from 60°C to 95°C. Gain was set to 10× for all runs. Data were acquired at the end of each cycle and each data point represented a mean of 20 fluorescence readings from each tube. Because of the Rotor Gene limited capacity (only 72 samples per run), samples were run for each of the full sibling groups (i.e., A, B, C or D) separately, but included all time points for 2 or 3 genes per run. Three replicates of every sample were run for each gene.

### Primer Specificity and Efficiency

Primer specificity was checked at the end of every run with melting curves for detection of any non-specific amplification or primer dimerization. Melting curves were obtained by heating from 60°C to 95°C at a rate of 0.1°C per second with continuous measurement of fluorescence. Data were checked using the analysis section (Melting A.FAM) of the Rotor Gene software.

In order to calculate the primer efficiencies (E), the dilution method was used on a pool of cDNA [33]. A dilution series was performed (1/1, 1/2, 1/5, and 1/10) on pooled cDNA from all samples. Data were obtained using the analysis section (Cycling A.FAM, Standard Curve) of the Rotor Gene software.

### Analysis

Ct values were obtained using the analysis section (Cycling A.FAM) of the Rotor Gene software. Noise slope correction was activated in order to take into account the background fluorescence level of every sample during the run. Also, dynamic tube normalization option was activated in order to determine the average background level of each individual sample before amplification commenced. Ct values were obtained after setting the threshold at 0.03 for all runs, and were then exported into Excel (Microsoft). Ct values were transformed in relative quantities (Q), using the delta Ct formula [34], and taking the first time point (per full sibling group) in the time series as a reference:

Q  =  E ^(refCt – sampleCt)^ with E being the amplification efficiency of the gene.

From relative quantity data, we were able to determine the best ICGs using GeNorm 3.5 (http://medgen.ugent.be~/jvdesomp/genorm/), for every full sibling group. Subsequently, normalization factors (NF) were calculated per sample:

NF  =  geometric mean (Q_ICG1_,Q_ICG2,_Q_ICG3_).

Finally, normalized expression (NE) levels were calculated per gene and per sample (normalization performed on averaged Ct values from technical replicates: qPCR triplicate runs).

NE  =  NF/Q_sample_^.^

Statistical analyses were performed for each full sibling group (biological replicates) and for each gene using Statistica 6.0 (StatSoft), testing first for data normality. After Log10 transformation, the normality assumption was validated and ANOVAs were performed on averaged values of technical replicates (triplicate qPCR runs) for the following factors: time (6 levels maximum), gene (4 levels maximum) and biological replicate (i.e., sample) which was nested within time but orthogonal with gene (i.e, sample(gene*time) in Statistica). A supplementary Post Hoc Tukey HSD test was performed if significant differences in gene expression were detected.

## Supporting Information

Figure S1 **Pooled gene expression results.** Mean (±SE) normalized expression levels per full sibling group for the genes A036-E7(black histograms), A049-E7 (dark grey histograms), apextrin (white histograms), and complement C3 (light grey histograms); from December 2007 to 6 months post-settlement (May 2008) for: **A**) full sibling group A; **B**) full sibling group B; **C**) full sibling group C; **D**) full sibling group D. Each bar represents the averaged normalized expression levels for all replicates (number of replicates written above each time point). Samples with low mRNA concentrations (<8.75 ng. µL^−1^) were not used and consequently some biological replicates (max n = 3/time point) and/or time points (max 6 time points) are missing from the analysis and the figure.
(EPS)
